# Prognostic Accuracy of DSM-5 Attenuated Psychotic Symptoms in Adolescents: Prospective Real-World 5-year Cohort Study

**DOI:** 10.1192/j.eurpsy.2022.511

**Published:** 2022-09-01

**Authors:** M. Iorio, C. Coci, E. Ballante, P. Fusar-Poli, R. Borgatti, M. Mensi

**Affiliations:** 1 University of Pavia, Department Of Brain And Behavioural Sciences, Pavia, Italy; 2 Università di Pavia e Bio data Center IRCCS Fondazione Mondino, Dipartimento Di Matematica, Pavia, Italy; 3 Institute of Psychiatry, Psychology & Neuroscience, King’s College London, Psychosis Studies, London, United Kingdom; 4 Fondazione Mondino - Istituto Neurologico Nazionale IRCCS, Child Neuropsychiatry, Pavia, Italy

**Keywords:** Psychosis, Adolescents, Attenuated Psychosis Syndrome, At risk of psychosis adolescents outcome

## Abstract

**Introduction:**

There is limited research in adolescent at risk for psychosis. The new criteria of Attenuated Psychosis Syndrome (APS) of Diagnostic and Statistical Manual of Mental Disorders- 5 (DSM-5) have not been validated.

**Objectives:**

The aims of this study were to: 1) characterize adolescent’s profile with APS (DSM-5 APS) compared to adolescents with early onset psychosis (EOP) and with other psychiatric disorders (non-APS); 2) to estimate their long-term risk of transition to psychosis and prognostic accuracy of DSM-5 APS.

**Methods:**

243 adolescents, aged 12-17, were included (October 2012- July 2019) and dived in three sub-groups (110 DSM-5 APS, 31 EOP, 102 non-APS). All underwent a comprehensive assessment evaluating: sociodemographic characteristics, family and personal history of any DSM-5 psychiatric disorders, psychopathological assessment and level of functioning. An annual follow-up evaluation was carried out (up to 7 years) including a clinical interview to investigate DSM-5 criteria for transition to psychosis.

**Results:**

DSM-5 APS adolescents had on average higher comorbid disorders (2.3) and intermediate psychopathological and functioning profile between non-APS/EOP. The cumulative risk of transition at 1,2,3, 4-5 years was 13%, 17%, 24.2%, 26.8% and 26.8% in DSM-5 APS group, 0%, 0%, 3.2%, 3.2% and 3.2% in the non-APS. The 5-year prognostic accuracy of the DSM-5 APS in adolescent was adequate (Area Under the Curve=0.77) with high sensitivity (91.3%) and suboptimal specificity (63.2%).

**Conclusions:**

The DSM-5 APS diagnosis can be used to detect help-seeking adolescents at risk of psychosis and predict their long-term outcomes, leading the way to new preventive approaches.

**Disclosure:**

The authors declare that they do not have a significant financial interest, consultancy or other relationship with products, manufacturer(s) of products or providers of services related to this abstrac.

